# Radiological and clinical differences among three assisted technologies in pedicle screw fixation of adult degenerative scoliosis

**DOI:** 10.1038/s41598-017-19054-7

**Published:** 2018-01-17

**Authors:** Yong Fan, Jin Peng Du, Ji Jun Liu, Jia Nan Zhang, Shi Chang Liu, Ding Jun Hao

**Affiliations:** 1 Department of Spine Surgery, Xi’an Jiao Tong University-affiliated Hong Hui Hospital, Youyidong Road, Xi’an City, 710000 China; 2 Medical College, Yan’an University, No 38 Guanghua Road, Yan’an City, 716000 Shaanxi Province China

## Abstract

The purpose of this study was to compare the clinical and radiological differences among three advanced guided technologies in adult degenerative scoliosis. A total of 1012 pedicle screws were inserted in 83 patients using a spine robot (group A), 886 screws were implanted in 75 patients using a drill guide template (group B), and 1276 screws were inserted in 109 patients using CT-based navigation (group C). Screw positions were evaluated using postoperative CT scans according to the Gertzbein and Robbins classification. Other relevant data were also collected. Perfect pedicle screw insertion (Grade A) accuracy in groups A, B, and C was 91.3%, 81.3%, and 84.1%, respectively. Clinically acceptable accuracy of screw implantation (Grades A + B) respectively was 96.0%, 90.6%, and 93.0%. Statistical analysis showed the perfect and clinically acceptable accuracy in group A was significant different compared with groups B and C. Group A exhibited the lowest intra-op radiation dose and group B showed the shortest surgical time compared with the other two groups. Robotic-assisted technology demonstrated significantly higher accuracy than the drill guide template or CT-based navigation systems for difficult screw implantations in adult degenerative scoliosis and reduced the intra-op radiation dose, although it failed to reduce surgery time.

## Introduction

Recently, major advances in biotechnology, such as image-guided, computer-assisted and robot-assisted systems, have been introduced in spine surgery, potentially resulting in better surgical outcomes with higher accuracy, smaller incisions, faster healing times, reduced trauma to normal tissues, and better pain relief^1,2^. In particular, the introduction of a robot-assisted pedicle screw fixation system (Renaissance™, Mazor Robotics, Caesarea, Israel, https://www.mazorrobotics.com/index.php), a spinous process-mounted miniature robot, holds great potential as an advanced idea, with a nearly 99% accuracy of clinically acceptable screw implantation. Though robotic-assisted surgery is an emerging field, similar to a baby making its first steps^3^, some superior features, such as minimal invasiveness and a high accuracy of screw implantation, are critical reasons why some “pioneering” surgeons are dedicated to this new field.

Other guided methods, such as patient-specific templates and CT-based navigation systems, provide increased accuracy compared with conventional fluoroscopy guided methods, as demonstrated by a series of low- to high-level studies^4,5^. These methods have been used for spine diseases such as fractures, degenerative diseases and so on, providing surgeons with visualization and guidance both preoperatively and intraoperatively in situations of normal spinal anatomy structures. However, it is unknown whether advanced Renaissance^TM^ will continue to maintain its advantages in adult degenerative scoliosis with difficult screw insertions when compared with patient-specific drill guide templates or image-guided navigation systems.

Adult degenerative scoliosis is a complicated spinal disorder, resulting from a combination of osteoporosis and asymmetric disc degeneration, with rotatory subluxation of multiple lumbar functional spinal units^6^. Prior characterization of vertebra deformities leads to difficult screw implantations; therefore, the purpose of this study was to investigate clinical and radiological differences between three advanced technologies in scoliosis by reviewing recorded information.

## Methods

### Subjects and study design

**Figure 1:**
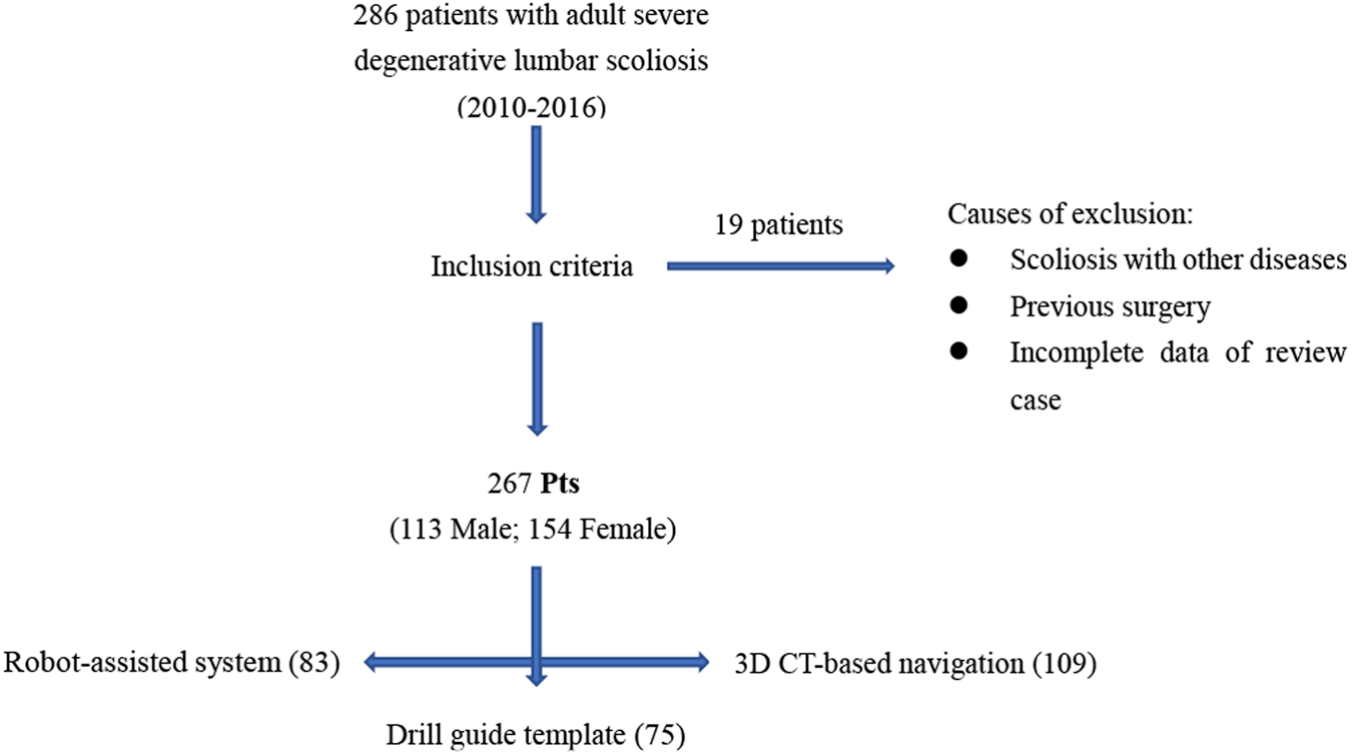
Diagram showing the process of patient selection.

A careful search of the Case Review Digital system of our hospital found that between 2009 and 2016, 286 patients were diagnosed with severe adult degenerative scoliosis. Patients meeting the following characteristics were eligible: (1) degenerative scoliosis often superimposed on a preexisting scoliosis with greater rotational deformity and a greater loss of lordosis; (2) imbalance in the coronal and sagittal planes; (3) Cobb angle >30°; and (4) obvious lower back pain and radiculopathy. Nineteen patients were excluded for several reasons, as follows: (1) degenerative scoliosis with a fracture, infection, tumor, discitis, or vertebral tuberculosis; (2) previous surgery; or (3) incomplete data in the review case. Finally, 3174 pedicle screws were inserted in 267 adult patients (113 male; 154 female) to reestablish both regional and global spinal balance in the frontal and sagittal planes and reconstruct three-column stability of the vertebral body (Fig. 1). Digital medical records, including demographic, clinical and diagnostic data, intraoperative and postoperative measurements, and radiology images, were retrospectively reviewed. Eighty-three patients underwent open robot-posterior lumbar interbody fusion (PLIF) surgery, 75 patients underwent patient-specific drill guide template-PLIF surgery, and 109 patients underwent CT-based navigation system-assisted PLIF surgery. All included patients underwent decompression and PLIF surgery for 1–3 fusion levels (Table 1). A total of 5 senior surgeons from the Department of Spine Surgery were qualified to perform intraoperatively demanding, assisted technology surgeries, and the decision-making process for choosing the technique for screw placement was based on the judgement of the senior surgeon. Hospital review board approval was obtained for all aspects of this study. Patients with severe degenerated scoliosis pathology that showed obvious clinical symptoms or neurological deficits were identified as candidates for surgical interventions for instrumentation, decompression and fusion.

**Table 1:** Detail baseline characteristics.

Groups				*P* value		
Parameter	Robot-PLIF	Template-PLIF	CT-based PLIF	RP vs TP	RP vs CP	TP vs CP
No. of patients	83	75	109			
Females, n (%)	48 (58)	41 (55)	65 (60)	0.689	0.802	0.503
Age (years)	61.6 ± 9.1	64.0 ± 7.7	63.9 ± 8.4	0.177	0.307	0.668
Mean BMI	25.8 ± 3.6	26.3 ± 4.2	27.3 ± 3.9	0.422	**<0.01**	0.099
No. of screws	1012	886	1276			
Mean no. of screws/case	12.2 ± 2.5	11.8 ± 2.3	11.7 ± 2.7	0.299	0.191	0.794
Mean no. of fixed segment	6.6 ± 1.5	6.8 ± 1.4	6.5 ± 1.7	0.389	0.672	0.209
Mean no of fusion level*	2.1 ± 0.6	2.1 ± 0.7	2.0 ± 0.7	1.000	0.299	0.342
**Fusion level (n)**						
one level	13	15	22	0.476	0.422	0.976
two levels	52	41	60	0.308	0.290	0.959
three levels	18	19	27	0.589	0.617	0.931
**Screw diameter (n)**						
6.5 mm	708	638	894	0.327	0.958	0.327
5.5 mm	94	62	144	0.070	0.120	**0.001**
**Upper-instrumented vertebra (n)**						
T9	3	2	9	0.734	0.188	0.116
T10	7	10	24	0.321	**0.011**	0.136
T11	19	12	30	0.276	0.466	0.067
T12	33	29	30	0.888	0.074	0.112
L1	21	22	16	0.570	0.065	**0.016**
**Lower-instrumented vertebra (n)**						
L3	8	10	13	0.465	0.615	0.777
L4	21	14	17	0.316	0.095	0.585
L5	41	32	44	0.397	0.212	0.756
S1	13	19	35	0.131	**0.009**	0.321

^*^No of fusion level mean the number of intervertebral space that require bone graft and the cage placement. RP: Robot-PLIF; TP: Drill guide Template-PLIF; CP: CT-based PLIF. Values that appear in boldface are statistically significant (p < 0.05).

### Robot-PLIF procedure

In the robot-PLIF cohort, preoperative computed tomography (CT) slice scans 1 mm thick were obtained for the 3D-reconstruction of the target level. CT data were transferred to a personal computer and imported in the Renaissance^TM^ planning software to design the screw trajectories, insertion points and implant sizes; these plans then were transferred to the Renaissance^TM^ workstation in the operating room. The preoperative CT scans were matched with intraoperative fluoroscopy images (anteroposterior and 60° oblique to the lateral plan marker X-rays) that were acquired of the patient’s anatomy for registration of the robot. Basic steps in the Renaissance^TM^ open-PLIF operation are summarized as follows: 1. preoperative planning; 2. posterior exposure and approach establishment; 3. attachment to the spinous process; 4. image acquisition and registration; 5. robot assembly and motion; 6. drilling and tubing system placement; 7. cannula introduction and K-wire placement; and 8. screw insertion. This was followed by decompression or posterior osteotomy for mobilization of the curves, interbody cage insertion and rod fixation after correction.

### Drill guide template-PLIF surgery procedure

The major procedures of patient-specific drill guide template assistance for screw implantation are as follows: 1. CT scanning with thin slices to target the spinal vertebral body; 2. reconstruction of captured data as a three-dimensional pattern to identify an optimal entry hole and calculation of the width, height and length of the target pedicle using special software; 3. design of a digital template contrary to the anatomical structure of the target level; 4. printing of the digital template, except a pre-reserved column, by a 3D-printer; 5. preoperative sterilization of the solid template; 6. Intraoperative clearance of the soft tissue behind the intended vertebral plate and spinous process; and 7. attachment of the template to the bone structure, followed by the insertion of screws through the pre-designed trajectory. Decompression and fusion procedures were performed, if necessary, along with a subsequent osteotomy for better scoliosis correction. More detailed surgical procedures have been described in a previous study^7^.

### CT-based PLIF surgery process

The assistance of a CT-based navigation system for surgery included 4 steps: 1. a reference clamp was attached to the spinous process of the vertebra; 2. intraoperative all-spine scanning; 3. obtained CT data were automatically matched in the navigation system; 4. fiberoptic markers were fixed on the navigation instrument; 5. the entry point was identified using a marked navigation probe; 6. screw trajectory was established during real-time visualization of the intraosseous position of the navigation tip; and 7. pedicle screw placement. If necessary, a subsequent osteotomy was performed for better scoliosis correction, followed by decompression and fusion, interbody cage insertion and rod fixation. More detailed surgical procedures have been described in a previous study^8^.

### Outcome measures

**Figure 2:**
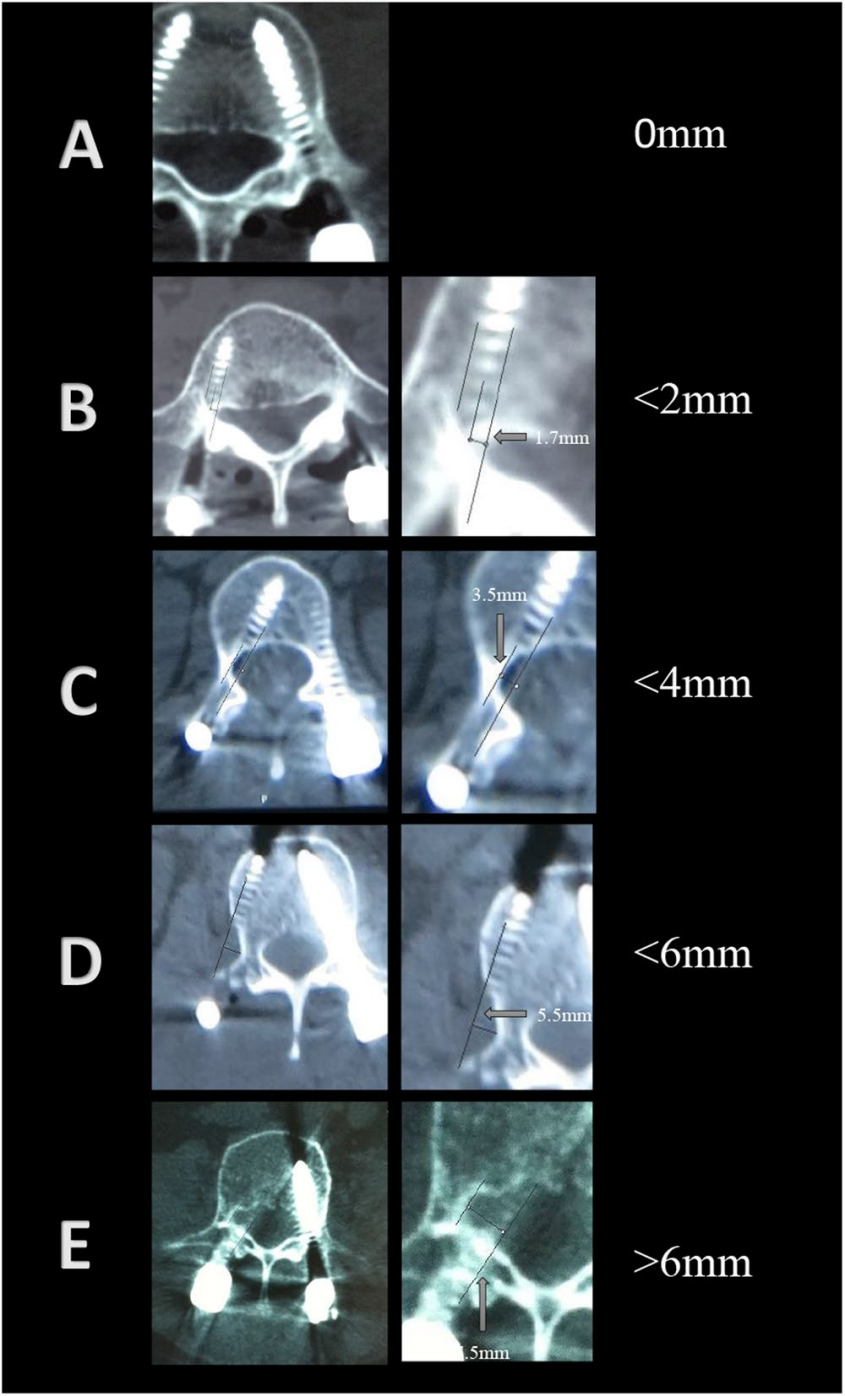
Gertzbein and Robbins classification scores are shown based on CT scans to reflect the deviation of the screw from the optimal trajectory. Grade (**A**): screw is completely within the pedicle; Grade (**B**), screw breaches the pedicle’s cortex by <2 mm; Grade (**C**), pedicle cortical breach <4 mm; Grade (**D**), pedicle cortical breach <6 mm; Grade (**E**), pedicle cortical breach >6 mm.

The primary measure was screw accuracy, which was assessed according to the Gertzbein and Robbins A to E classification system^9^ using axial, coronal and sagittal reconstruction views of the CT scans (Fig. 2). The grading system included the following levels: Grade A: the screw is completely within the pedicle; Grade B, the screw breaches the pedicle’s cortex by <2 mm; Grade C, pedicle cortical breach <4 mm; Grade D, pedicle cortical breach <6 mm; and Grade E, pedicle cortical breach >6 mm. Meanwhile, Grade R means a screw trajectory was proposed by the guidance device but had to be revised manually; therefore, such intraoperatively revised screw placements were considered to be inaccurate. Two spine surgeons (Yong Fan, Jin Peng Du) independently evaluated all postoperative CT scans blind to three groups, where screws graded A were “perfect”, those graded A + B were “clinically acceptable”, and those graded C-E had a significant deviation from the intended trajectory and were also considered to be inaccurate.

Other secondary radiological measurements were also collected, such as proximal facet joint violations, the orientation of pedicle encroachment and intra-op fluoroscopic dose (mSv). Scoliosis correction parameters were assessed with plain radiographs preoperatively and postoperatively, which included measurements of the coronal plane deformity according to the Cobb method and the evaluation of spinopelvic parameters such as pelvic tilt (PT), lumbar lordosis (LL), and sacral slope (SS). We also collected the time of surgery, adverse events, cases of revision surgeries and intraoperative blood loss.

### Statistical analysis

Categorical and continuous variables were respectively analyzed by ANOVA and SNK tests. Mean values are presented as the mean ± SD. All statistical analyses were performed using the SPSS 20.0.0 statistics package (SPSS, Inc., Chicago, IL, USA), with an alpha level of significance set at 0.05.

## Results

### Baseline characteristics

The mean patient age was 63.2 years (range: 45–80), and the gender ratio (m/f) was 113/154. The average BMI was 26.5. The mean BMI between group A and group C, the number of 5.5 mm screws between group B and group C and the number of upper or lower instrumented vertebra between three groups showed significant differences. The rest of the baseline parameters did not significantly differ between the 3 groups. The screw diameter varied from 5.5 to 6.5 mm, and the fusion level varied from 1–3 levels. Detailed parameters are shown in Table 1.

### Accuracy of pedicle screw placement

As shown in Table 2, “perfect” accuracy of pedicle screw insertion (Grade A) in groups A, B, and C was 91.3%, 81.3%, and 84.1%, respectively. “clinically acceptable” screw implantation (Grade A + B) accuracy in groups A, B, and C was 96.0%, 90.6%, and 93.0%, respectively. Possible breaches (Grades C + E) respectively were 3.4%, 6.0%, and 5.5%; all primary endpoints are summarized as a column graph in Supplementary Figure S1. The rate of intraoperative screw revisions was 0.6%, 3.4%, and 1.6%, respectively, which were revised because the screw did not have sufficient bone grip or a lateral fluoroscopy showed the entry point was not pointing directly on the craniocaudal center of the pedicle; a new trajectory then had to be re-identified based on open fluoroscopy guidance. Therefore, every revised screw was considered to be inaccurate and clinically unacceptable. Statistical analysis showed group A had statistically significant superiority (P < 0.05) in “perfect and clinically acceptable” accuracy compared with groups B and C, but there was no significant difference in the “perfect” accuracy between group B and group C, while group C seemed to exhibit higher “clinically acceptable” accuracy than group B (P < 0.05). Meanwhile, the robot-PLIF group exhibited the lowest rate of manual screw revisions compared other two groups, while the drill guide template-PLIF group showed the highest.

**Table 2:** Accuracy of pedicle screw placement among three assisted technologies.

Screw Position*	Robot-PLIF (n [%])	Template-PLIF (n [%])	CT-based PLIF (n [%])	*P* value		
				RP vs TP	RP vs CP	TP vs CP
A	924 (91.3)	720 (81.3)	1073(84.1)	**<0.001**	**<0.001**	0.062
B	48 (4.7)	83 (9.4)	114 (8.9)	**<0.001**	**<0.001**	0.730
A + B	972 (96.0)	803(90.6)	1187(93.0)	**<0.001**	**0.019**	**0.043**
C	28 (2.8)	33 (3.7)	56 (4.4)	0.238	**0.040**	0.445
D	6 (0.6)	14 (1.6)	11 (0.9)	**0.036**	0.456	0.125
E	0 (0.0)	6 (0.7)	2 (0.2)	**0.009**	0.208	0.050
R	6 (0.6)	30 (3.4)	20 (1.6)	**<0.001**	**0.029**	**0.006**
Total	1012	886	1276			

*Screw position identified according to Gertzbein and Robbins A to E classification; RP: Robot-PLIF; TP: Drill guide Template-PLIF; CP: CT-based PLIF. Values that appear in boldface are statistically significant (p < 0.05). Grade “R” means some screws trajectory were proposed by the guided device but had to be revised manually.

### Secondary results

Facet joint violations were evaluated according to the classification described by Kim *et al*.^10^ (Table 3), where Grade 0 (group A-C: 98.9%, 98.0%, 97.6%), Grade 1 (group A-C: 1.1%, 1.8%, 2.1%), and Grade 2 (group 1–3: 0%, 0.2%, 0.3%) did not show a significant difference between groups (P > 0.05). The robot-PLIF group showed fewer hazardous orientations because the orientations of pedicle encroachment were all integrated into a lateral approach; however, the other groups exhibited pedicle encroachment in four orientations.

**Table 3:** Secondary radiological results.

	Robot-PLIF	Template-PLIF	CT-based PLIF
Proximal facet joint violation*			
Grade 0	1001 (98.9%)	868 (98.0%)	1245 (97.6%)
Grade 1	11 (1.1%)	16 (1.8%)	27 (2.1%)
Grade 2	0 (0)	2 (0.2%)	4 (0.3%)
**Orientation of pedicle encroachment**			
Medial	0	2	1
Lateral	88	72	76
Superior	0	4	7
Inferior	0	5	5

*Facet joint violation was evaluated according to the classification described by Kim *et al*. Grade 0 = no impingement, Grade 1 = screw head in contact/suspected to be in contact with facet joint, Grade 2 = screw clearly invaded the facet joint.

In the scoliosis-relevant parameter measures, as shown in Table 4, there were no differences in changes in the Cobb angle value, PT, LL or SS between the three groups, except in group B vs. group C in changes in the Cobb angle value (P = 0.02).

**Table 4:** Measurements of scoliosis correction by X-ray plain film.

	Cobb (°)				LL (°)				SS (°)				PT (°)			
	Pre	Post	*p*	**Δ**	Pre	Post	*p*	**Δ**	Pre	Post	*p*	△	Pre	Post	*p*	**Δ**
Robot-PLIF (n = 83)	46 ± 8	11 ± 7	**<0.001**	36 ± 7	23 ± 11	54 ± 6	**<0.001**	32 ± 9	11 ± 6	40 ± 6	**<0.001**	30 ± 5	28 ± 3	24 ± 4	**<0.001**	4 ± 4
Template-PLIF (n = 75)	44 ± 9	10 ± 8	**<0.001**	35 ± 9	25 ± 10	56 ± 8	**<0.001**	31 ± 8	10 ± 6	39 ± 7	**<0.001**	29 ± 7	29 ± 4	25 ± 3	**<0.001**	5 ± 4
CT-based PLIF (n = 109)	49 ± 9	12 ± 9	**<0.001**	38 ± 8	22 ± 13	56 ± 9	**<0.001**	33 ± 8	10 ± 5	41 ± 6	**<0.001**	31 ± 7	28 ± 4	24 ± 4	**<0.001**	4 ± 3
*p* (RP VS TP)	0.14	0.37	—	0.4	0.24	0.08	—	0.14	0.3	0.34	—	0.26	0.08	0.08	—	0.12
*p* (RP VS CP)	**0.01**	0.4	—	0.07	0.57	0.07	—	0.41	0.21	0.25	—	0.22	1	1	—	1
*p* (TP VS CP)	**<0.001**	0.12	—	**0.02**	0.08	0.07	—	0.1	1	**0.04**	—	0.06	0.1	**0.05**	—	0.07

RP: Robot-PLIF; TP: Drill guide Template-PLIF; CP: CT-based PLIF; **Δ** = |Post-Pre|, Cobb: Cobb angle; LL**:** lumbar lordosis angle; SS**:** Sacral slope; PL**:** Pelvic tilt; Bold values are statistically significant (P < 0.05).

**Figure 3:**
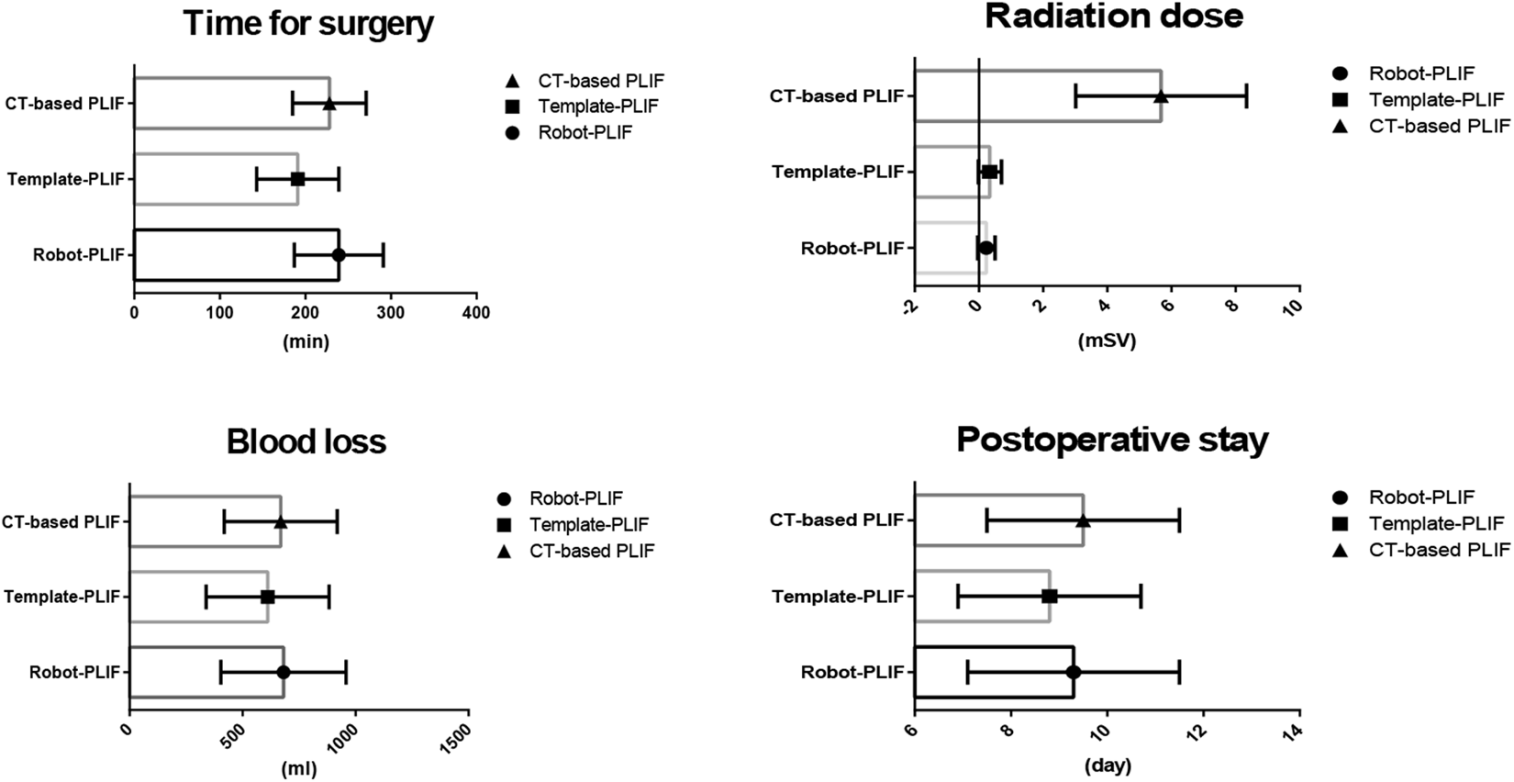
Column graph of the comparisons of time of surgery, radiation dose, blood loss and postoperative stay between the three assistive technologies.

The overall surgical times from skin to skin are shown in Table 5 for group A (239 ± 52 minutes), group B (191 ± 48 minutes) and group C (228 ± 43 minutes). Clearly, group A showed more surgical time than group B but was not significantly different from group C. The prevalence of total adverse events in group A (6 [7.23%]) showed no difference compared with group B (9 [12.00%]) or group C (9 [8.23%]), as well as dural tears, surgical wound revisions, wound infections and neurological complications. Regarding intra-op radiation dose (group A-C: 0.41 ± 0.39 mSV; 0.34 ± 0.36 mSV; 5.68 ± 2.66 mSV), the robot-PLIF group showed less than the CT-based PLIF group (P < 0.05) but was not different from the template-PLIF group. Blood loss (group A-C: 681 ± 277 ml; 611 ± 272 ml; 669 ± 250 ml) and postoperative stay (group A-C: 9.3 ± 2.2 d; 8.8 ± 1.9 d; 9.5 ± 2.0 d) demonstrated no significant differences between the three groups. Several cases required postoperative revision surgeries, mainly for cage dislodgement or screw malpositioning, but there were no differences in the revised rate between groups. Some clinical results of the continuous variables are integrated into four column graphs shown in Fig. 3.

**Table 5:** Secondary endpoints for clinical outcomes.

	Robot-PLIF	Template-PLIF	CT-based PLIF	*P* value		
				RP vs TP	RP vs CP	TP vs CP
Time for surgery (min)	239 ± 52	191 ± 48	228 ± 43	**<0.001**	0.111	**<0.001**
Intra-op radiation dose (mSv)	0.41 ± 0.39	0.34 ± 0.36	5.68 ± 2.66	0.244	**<0.001**	**<0.001**
Blood loss (ml)	681 ± 277	611 ± 272	669 ± 250	0.112	0.754	0.138
Postoperative stay (d)	9.3 ± 2.2	8.8 ± 1.9	9.5 ± 2.0	0.130	0.512	**0.018**
**Revision surgery (n)**						
Cage dislodgement	2	1	2	0.621	0.782	0.792
Screw malposition	0	3	2	0.066	0.215	0.375
Total	2	4	4	0.337	0.619	0.587
**Adverse events (n)**						
Dural tears	1	2	1	0.494	0.846	0.351
Surgical wound revision	4	5	6	0.617	0.832	0.744
Wound Infections	1	1	1	0.942	0.846	0.789
Neurological complications	0	1	1	0.291	0.382	0.789
Total	6 (7.23%)	9 (12.00%)	9 (8.23%)	0.445	0.793	0.579

RP: Robot-PLIF; TP: Drill guide Template-PLIF; CP: CT-based PLIF; Bold values are statistically significant (P < 0.05).

## Discussion

Adult scoliosis is a complex three-dimensional deformity of the spine that is the result of a progressive, coupled, asymmetrical degeneration of the intervertebral discs and facet joint complexes^11^. In addition, some patients may undergo both an asymmetrical collapse of the vertebral bodies and lateral slippage, subsequently developing multisegmental involvement with sagittal and coronal unbalance. Moreover, severe adult scoliosis is often superimposed on preexisting scoliosis, with greater rotational deformity and greater loss of lordosis^12^. It always requires surgical treatment with long-level pedicle screw instrumentation with posterior lumbar interbody fusion; however, the complex anatomical structures of deformed vertebra add extra difficulty in screw placement, which prevents the first step of spine balance reconstruction. Therefore, many advanced technologies were developed to focus on this problem.

The Renaissance^TM^ system-based accurate auxiliary measures were designed to ensure the safety and accuracy of pedicle screw insertion, although this is doubted by experienced Chinese doctors who are good at free-hand screw insertion, mainly because of the high confidence in their technique or a dislike for complicated procedures. However, drill guide templates and CT-based navigation have been confirmed to have sufficient safety and accuracy levels for spine surgery by several high-level clinical studies^10,13,14^.

A retrospective series study by Devito summarized the first experiences with the Renaissance^TM^ robot from 14 spine centers worldwide and found a 98% rate of clinically acceptable screw insertion, with no permanent nerve damage occurring. However, 9% of the screws showed a minor pedicle breach, which may still have excellent biomechanical properties without the potential of clinically apparent neurological or vascular impairment, which can surely be evaluated as safe, clinically acceptable malpositioning. Another strong statement was offered by Hyun^15^, who designed a prospective randomized clinical trial between minimally invasive robotic- and open fluoroscopic-guided spinal instrumented fusions. Pedicle screw positions were classified using a modification of the Gertzbein-Robbins scale, and 97.7% of screws were completely within the pedicle, although 2.3% of screws breached the pedicle’s cortex by <2 mm; all screws were clinically acceptable without screw-related neurological damage.

Whether spine robot-guidance continues to maintain its advantages in open surgery for adult degenerative scoliosis with difficult screw insertions compared to patient-specific drill guide templates or image-guided navigation system is an interesting question demanding research. Certainly, other factors, such as surgical time, intra-op radiation dose, adverse events, scoliosis correction outcomes, blood loss, and length of day, must also be comprehensively considered as important attentional points.

In our review, Renaissance^TM^ succeeded in showing a sufficient difference in terms of the accuracy of “perfect” and “clinically acceptable” pedicle screw insertions compared with the other two techniques. High accuracy of pedicle screw placements associated with low risk of revision surgery due to the screw relevant neurological complications. In total, 96.0% of the screws (Grade A + Grade B) in the robot-PLIF group in this study were considered to be “clinically acceptable” by us, less than the 98% reviewed by Devito because he identified pedicle cortical breaches <4 mm without neurological complications as clinically acceptable accuracy; on the other hand, he used the robot technique only for normal lumbar pedicle screw insertion. Strictly speaking, a screw that breaches the pedicle’s cortex by ≥2 mm is subject to a risk of long-term screw-related neurological complications. In our experience, we only classify Grade A + B as an appropriate accuracy criterion for “clinically acceptable” screw placement. Interestingly, the accuracy of perfect placements in the drill guide template group (81.3%) was not significantly different compared with the robot-assisted group (84.1%), possibly because, during the last decade, gradual advances in computer and additive manufacturing technologies have resulted in a more practical and sophisticated application of individual templates in spine surgery, with hopeful outcomes^16^. Otherwise, three-dimensional fluoroscopic image guidance systems demonstrated a significantly higher pedicle screw placement accuracy than CT-based navigation system and 2D fluoroscopic image guidance methods, what is the result when it is compared with the spine robot, this is a subject worth further studying^4,17^.

We also wonder if an association exists between the accuracy of screw placement and results of scoliosis correction, but it seems that differences in accuracy did not lead to variations in Cobb angle, PT, LL or SS. Though Group B showed differences in the changes of the Cobb angle values compared to Group C, this may because the preoperative Cobb angles were not aligned. Proximal facet joint violations still failed to be identified as different between the three groups, and it is unclear that if this situation results from the high accuracy of screw insertions.

As shown in Fig. 3, the robot-assisted method and CT-based navigation required more time for surgery from skin to skin, and this may result from their complicated procedures. There was the point that the routine use of some systems may further reduce the operating time, but current systems do not meet spine surgeon’s expectations in terms of ease of use and integration into the surgical work flow, factors limited the routine use include the lack of equipment, inadequate training, and high costs^18^. We also found this issue in the research process, but due to the above mentioned reasons for these systems, the routine usage rate is relatively low in our health care, system need continuous improvement to increase its use. The robot-PLIF group showed a small intra-op radiation dose that was similar to the template-PLIF group, and they all demand fluoroscopy for preoperative location and postoperative confirmation of the placement of every screw as well as the extra fluoroscopy needed for matching in the robotic process^19,20^. Regarding adverse events, blood loss, revision surgery and length of stay, the open robot technique failed to show an advantage over the other two techniques, but the lowest rate of intraoperative revision of screws occurred in the robot-PLIF; surely, the high accuracy of screw implantation reduces screw revisions due to malpositioning. Revised screws were included in the analysis as poor screw positioning that was clinically unacceptable.

The Renaissance^TM^ platform uses a computerized mechanical positioning system that assists surgeons in inserting implants along the planned trajectory. There are two advantages; first, it requires registration without using bony landmarks that can be applied even in difficult insertions and deformities of the spine. Second, the robot system works without relying on a camera tracking mechanism and avoids all the problems of reconnaissance between cameras and navigation instruments or targets^21^.

Drawbacks in the process of the robotic system usage include a few patients with severe degenerative scoliosis who were operated on but failed to match the preoperative CT scanning with the intraoperative fluoroscopy images; some time was wasted, and the surgeon had to change the surgical technique to free hand. Involving all search processes from the Case Review Digital system, 8 such cases occurred. Although there is good registration, there could still be a possible phenomenon where the cannula slides off an angled bone surface, resulting in difficult-to-prevent lateral screw inaccuracies^22^. Normally, when this occurs lateral to the facet joint, it demands careful attention when using the robot. In addition, the relatively expensive costs for the robot’s supplies and maintenance add an extra financial burden to patients.

Two limitations of our study should be mentioned. The first limitation is that patient characteristics, such as mean BMI, did not align between group A and group C, and a similar condition occurred with screw diameter and upper/lower instrumented vertebra. Another limitation is that all studies derived from a database that included four spine surgeries, nearly 40 surgeons participated in the employment of the three assistive tools, and the variances in the proficiency of tool employment and surgical skills may add a slight bias to the primary or secondary endpoints. Therefore, additional prospective, randomized, controlled studies are required.

## Conclusions

Robotic-assisted technology showed obviously higher accuracy than drill guide template or CT-based navigation systems for difficult screw implantations in adult degenerative scoliosis; the method also reduced intra-op radiation dose, but it did not reduce time for surgery.

## Electronic supplementary material

Supplementary Figure S1
