# Supplementary material for: Radiological and clinical differences among three assisted technologies in pedicle screw fixation of adult degenerative scoliosis
Source: Sci Rep. 2018 Jan 17;8:890. doi: 10.1038/s41598-017-19054-7 (PMC5772356; doi:10.1038/s41598-017-19054-7)
Supplement: Supplementary file 1 — Supplementary Figure S1 [file 41598_2017_19054_MOESM1_ESM.pdf]

Radiological and clinical differences among three assisted technologies in pedicle screw fixation of adult degenerative scoliosis

Yong Fan[1,3] ▪ **Jin Peng Du**[1,2,3] ▪ **Ji Jun Liu**[1] ▪ **Jia Nan Zhang**[1] ▪ **Shi Chang Liu**[1] ▪ **Ding Jun Hao**[1,2]

[3] Yong Fan and Jin Peng Du both considered as the first author

[1]Department of Spine Surgery, Xi'an Jiao Tong University-affiliated Hong Hui Hospital, Youyidong Road, Xi'an City 710000, China.

[2]Medical College, Yan'an University, No 38 Guanghua Road, Yan'an City 716000, Shaanxi Province, China.

Corresponding Author: Ding Jun Hao, Email: hhyyhdj@126.com, Tel/Fax: 0086-29-87800002.

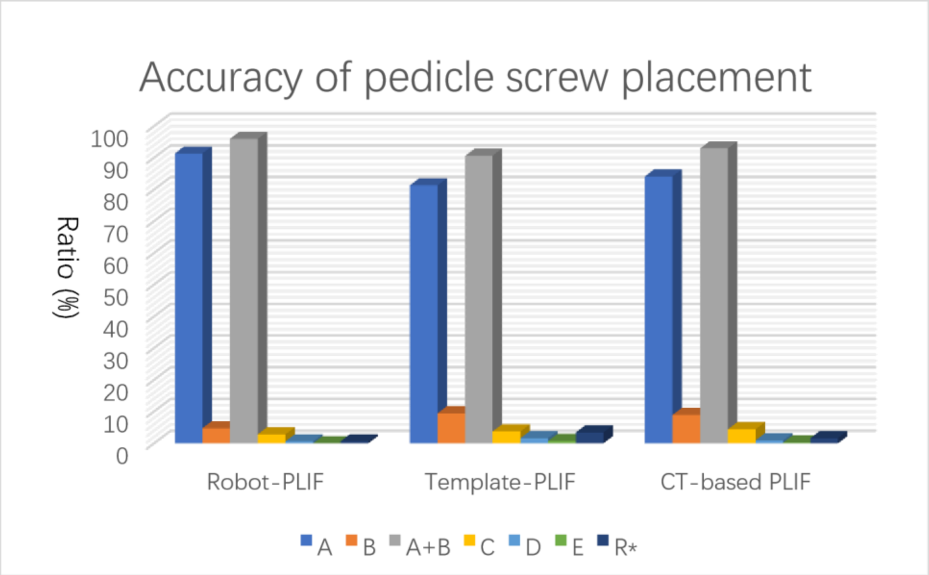

Supplementary Figure S1 Column graph of the comparisons of time of surgery, radiation dose, blood loss and postoperative stay between the three assistive technologies.
